# The Prevalence of Undiagnosed Ptosis Among Candidates for Upper Eyelid Blepharoplasty

**DOI:** 10.1093/asjof/ojad079

**Published:** 2023-09-01

**Authors:** Bahram Eshraghi, Mohsen Pourazizi, Akbar Taghian, Samira Chaibakhsh, Ali Aghajani

## Abstract

**Background:**

Because upper eyelid blepharoplasty has become a popular aesthetic facial surgery, surgeons should be aware that age-related changes in the eyelid are not confined to skin laxity and orbital fat prolapse.

**Objectives:**

This study was designed to assess the prevalence of undiagnosed ptosis among blepharoplasty candidates as one of the causes of unsatisfactory surgical results.

**Methods:**

From December 2018 to December 2022, blepharoplasty candidates were meticulously assessed for their upper eyelid and eyebrow position. Patients who were aware of their ptosis were excluded, and the other patients were classified as mild, moderate, or severe ptotic based on margin reflex distance 1. The eyebrow height was also assessed in the mid-pupillary line to assess the relationship between the severity of ptosis and eyebrow asymmetry.

**Results:**

The authors found that 13.7% of the 2530 blepharoplasty candidates in this study had undiagnosed ptosis. Most of these patients had mild ptosis (85.5%), and they were significantly older than nonptotic patients. The rate of prevalence of ptosis was significantly higher in patients with eyebrow asymmetry (75.3% vs 3.7%); however, the severity of ptosis was not associated with the severity of eyebrow asymmetry.

**Conclusions:**

Ptosis should be cautiously looked for and addressed for treatment in candidates for upper blepharoplasty. In most patients with masked ptosis, the severity of eyelid drooping is mild and could remain undiagnosed until after the surgery and cause unsatisfactory aesthetic results. The presence of eyebrow asymmetry could be a key feature to unmask an undiagnosed ptosis.

**Level of Evidence: 3:**

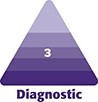

Aging causes various changes in the eyelids, including dermatochalasia, dynamic wrinkling, hyper pigmentation, and blepharoptosis.^1^ The last-mentioned condition, blepharoptosis, is defined as a reduction of palpebral fissure height to various degrees. Upper eyelid blepharoplasty is a commonly performed aesthetic procedure, particularly among middle-aged females.^2^ Surgeons should take into consideration the fact that age-related changes in the eyelids extend beyond the increased skin and puffiness. It is important to carefully consider the presence of other issues associated with dermatochalasis, such as blepharoptosis, when planning for surgery.

Although some candidates for upper eyelid blepharoplasty may exhibit an obvious droopy eyelid with a significant decrease in margin reflex distance 1 (MRD1), detecting droopy eyelids can be more challenging in others. This is especially true for patients with mild-to-moderate blepharoptosis, in which upper eyelid puffiness can cause pseudoptosis, making it difficult to distinguish it from true ptosis. Most of these patients are primarily concerned about their overhanging eyelid skin and are not aware of the droopy eyelid that is obscured behind it. Failure to detect and address this underlying ptosis can result in unsatisfactory aesthetic outcomes and may even be perceived as a surgical complication by the patient. Therefore, detailed history-taking and careful eye examination are necessary for accurate surgical planning. Surgeons should look for signs that indicate the presence of undiagnosed ptosis before proceeding with surgery. Some of the most important indicators of undiagnosed ptosis, particularly in unilateral cases, are higher eyebrow height (eyebrow asymmetry), a high supratarsal crease, and a deeper forehead crease on the side of ptosis leading to facial asymmetry.^3^

In a study by Hashemi et al,^4^ the frequency of ptosis was reported to be ∼5%, with a tendency to increase with age. In this study, the signs of masked ptosis, such as the position of the eyebrows and forehead crease, were not investigated. In another study, which was done by Abdolalizadeh et al,^5^ it was found that there was an even higher prevalence of ptosis in candidates for upper eyelid blepharoplasty. In this retrospective study on 1400 candidates for upper eyelid blepharoplasty, the overall prevalence rate of masked and unmasked ptosis, determined using direct (MRD1) and indirect (eyebrow asymmetry, etc.) signs, was 46.6%, with masked ptosis accounting for 31% and unmasked ptosis accounting for 15.6%.

In our opinion, facial plastic surgeons should be aware of the prevalence of undetected ptosis in patients seeking upper eyelid cosmetic surgery. Therefore, this study was designed to determine the prevalence of static masked ptosis in candidates for upper eyelid blepharoplasty and its association with eyebrow asymmetry.

## METHODS

This retrospective study was carried out between December 2018 and December 2022 at the oculoplastic clinic of the Feiz Hospital and private office (B.E.), Isfahan, Iran. The study was approved by the Ethics Committee of the Isfahan University of Medical Sciences (code: IR.IUMS.REC.1399.880), and all investigations adhered to the tenets of the Declaration of Helsinki. The medical records of the candidates for upper eyelid blepharoplasty were studied to obtain information on them and their preoperative data. Informed consent was obtained from the participants of this study.

Patients were excluded if they had a history of periocular botulinum toxin injection in the past 6 months, or any disease affecting the eyelid position such as thyroid eye disease, facial nerve palsy, facial or blepharospasm, and eyelid mass (lesion). Additionally, patients with a history of previous eyelid surgery were excluded from the study.

In this study, patients who were aware of their eyelid ptosis (unilateral or bilateral) and sought correction for both ptosis and dermatochalasis were also excluded. In the remaining patients, the eyelid position was evaluated, and palpebral fissure height (PFH) and MRD1 were recorded as the distance (millimeter) between the upper eyelid margin and the lower eyelid margin and corneal light reflex in the primary gaze position with 0.5 mm increment, respectively (Figure); if the overhanging eyelid skin hampered MRD1 measurement, it was gently held up by the senior author (B.E.) to facilitate the measurement and omit patients with pseudoptosis. A patient was marked as ptotic if 1 eyelid was located >0.5 mm below the other one. Mild, moderate, and severe unilateral ptoses were defined as the intereye difference of PFH between 0.5 and 2 mm, 2 and 4 mm, and >4 mm, respectively. Bilateral eyelid ptosis was defined as MRD1 <3 mm in both eyes; it was graded as mild, moderate, and severe if MRD1 was between 2 and 3 mm, 0 and 2 mm, and <0 mm, respectively.

**Figure.**
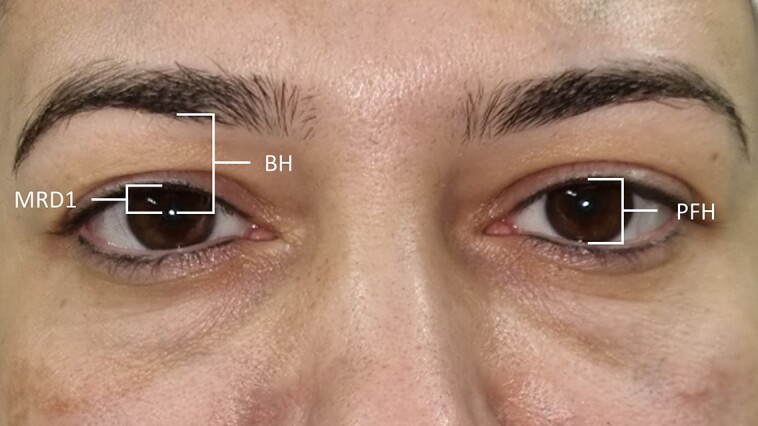
Depiction of the measured parameters on the eyelid of a 43-year-old female candidate for upper eyelid blepharoplasty. BH, brow height; MRD1: margin reflex distance 1; PFH, palpebral fissure height.

Eyebrows were considered asymmetric if the higher margin of 1 eyebrow was placed at least 1 mm above the other in the mid-pupillary line in primary gaze position.^6^ If the difference exceeded 1 mm, the eyebrows were considered asymmetric, and if it exceeded 3 mm, they were classified as severely asymmetric.

### Statistical Analysis

Mean and standard deviation were reported to describe continuous variables. The categorical variables were described in terms of frequency and percentage. A *t* test was performed to compare the mean of continuous variables, and a χ^2^ test was done to assess the relationship between categorical variables. A value <0.05 was considered significant. All analyses were done by using SPSS 24 (IBM; Armonk, NY).

## RESULTS

Overall, 2530 patients were included in this study; 64 (2.5%) of them were male and 2466 (97.5%) were female. The mean age of the participants was 47.56 ± 11.14 years (range: 22-82 years). Male candidates for upper blepharoplasty were older than females (48.69 ± 11.13 vs 47.53 ± 11.14); however, the age difference was not significant (*P* = .414). The overall rate of prevalence of ptosis in the study population was 13.7% (346 out of 2530 patients; 0.9% of them were male and 99.1% were female). A percentage of 4.7 males and 13.9 females had ptosis; the difference between the prevalence of ptosis in males and females was statistically significant (*P* = .045, Table 1).

**Table 1.** Characteristics of Blepharoplasty Patients With and Without Eyelid Ptosis

Characteristic		*n*	Without ptosis	With ptosis	*P*-value
Age		2530	47.34 ± 11.11 (range: 20-83)	48.95 ± 11.25 (range: 21-73)	.013^a^
Gender	Male	64	61 (95.3%)	3 (4.7%)	.045^b^
	Female	2466	2123 (86.1%)	343 (13.9%)	
Eyebrow asymmetry	+	352	87 (24.7%)	265 (75.3%)	<.001^b^
	−	2178	2097(96.3%)	81 (3.7%)	

a *t* test. ^b^χ^2^ test.

The mean MRD1 in patients with ptosis was 3.7 mm. Among the patients with ptosis, 296 (85.5%) of them had mild eyelid ptosis and 50 (14.5%) of them had moderate eyelid ptosis (Table 2). The mean age of patients with ptosis was significantly higher than those without ptosis (*P* = .013, Table 1).

**Table 2.** The Prevalence of Ptosis in Patients With Different Eyebrow Asymmetries

Characteristic		*n*	Ptosis		*P*-value
			Mild	Moderate	
Eyebrow asymmetry	Mild	220	188 (85.5%)	32 (14.5%)	.099
	Severe	45	35 (77.8%)	10 (22.2%)	
Without eyebrow asymmetry		81	73 (91.2%)	8 (8.8%)	

Eyebrow asymmetry was found in 352 out of 2530 (13.9%) participants of this study. The prevalence rate of ptosis in patients with eyebrow asymmetry was significantly higher than those without eyebrow asymmetry (75.3% vs 3.7%, *P* < .001, Table 1). We also evaluated the prevalence of ptosis among the 352 patients with eyebrow asymmetry. Overall, 265 out of 352 patients with eyebrow asymmetry had ptosis. A total of 220 out of 296 (74.1%) patients with mild eyebrow asymmetry and 45 out of 56 (80.4%) of patients with severe eyebrow asymmetry had ptosis. The difference between the prevalence of ptosis in patients with mild and severe eyebrow asymmetry was not statistically significant (*P* = .319).

On the other hand, among the patients with ptosis, 76.6% (265 out of 346, Table 2) had eyebrow asymmetry. The mean age of ptotic patients with eyebrow asymmetry was 48.91 ± 11.35 and the age of ptotic patients without asymmetry was 49.9 ± 10.97 years. The difference between them was not significant (*P* = .899). Table 2 shows that the prevalence rates of moderate ptosis in patients with mild and severe eyebrow asymmetry are 14.5% and 22.2%, respectively; the difference between the 2 groups was not statistically significant (*P* = .099, Table 2).

There were only 11 patients (10 females and 1 male) who had undiagnosed bilateral ptosis. The mean age of these patients was 59.3 ± 6.75 years, which was significantly higher than that of patients with unilateral ptosis (*P* = .041). Nine patients had mild bilateral ptosis, 1 patient had moderate bilateral ptosis, and 1 patient had severe bilateral ptosis. None of these patients had eyebrow asymmetry.

## DISCUSSION

In this study, it was revealed that up to 13% of candidates for upper eyelid blepharoplasty had undiagnosed ptosis. Age-related changes in the eyelid can manifest as eyelid laxity, dynamic wrinkles, and hyperpigmentation.^1^ These unwelcome features encourage such patients to seek restoration of their youthful eyelid appearance, thus triggering a tremendous demand for eyelid cosmetic surgery.^7^ However, aesthetic surgeons should be aware that the senile changes in the periorbital area extend beyond the eyelid skin. Decreasing the length and the width of eyelid fissure, orbital septum pseudoherniation, ptosis of the lacrimal glands, orbital fat atrophy, and bony remodeling of the orbit are some of the other senile periocular changes.^8,9^ Eyelid ptosis is one of the age-related eyelid changes that can be masked by dermatochalasia and may go unnoticed until after the blepharoplasty procedure. Loss of orbital fat and orbital bony remodeling and the aging enophthalmos that follows, in combination with levator aponeurosis degeneration, are the main causes of involutional eyelid drooping in the elderly.^1^ The gradual loss of the youthful eyelid appearance causes problems, specifically the droopy eyelid, which blepharoplasty candidates are generally unaware of. There are multiple ways of addressing this problem alongside blepharoplasty,^10^ and leaving this issue unattended by the aesthetic surgeon would yield unsatisfactory surgical results. The mean MRD1 of ptotic patients of this study was 3.7 mm, but >85% of them had mild ptosis (<2 mm), meaning that it could be easily missed by an untrained eye and should therefore be meticulously sought for.

In order to unmask this problem, attention should be paid to concomitant findings. Enhanced forehead rhytids and facial asymmetry may be the aesthetic concerns that are primarily noted in these individuals. In our study, we found that eyebrow elevation was present in 76.6% of patients with masked ptosis. In candidates for blepharoplasty, some asymmetry in periocular measurements could be found in up to 93% of them, with central and temporal eyelid and brow asymmetry being the mostly observed conditions. However, eyebrow elevation, especially in unilateral cases, could indicate an underlying dysfunction. The cause of this asymmetric or unbalanced periocular muscular activity has been discussed by many authors. In general, the cause of this asymmetry could be classified as primary asymmetric eyebrow elevation and secondary (or compensatory) eyebrow elevation as a result of visual axis occlusion. In compensatory eyebrow elevation, the frontalis muscle contracts to keep the upper eyelid out of the way. Brow elevation on the side of the ptotic eye is a well-known finding. In a study by Thorne et al,^11^ it was revealed that ∼25% of ptotic patients had brow height asymmetry. In our study, however, we found that >75% of ptotic patients had asymmetry caused by an elevated brow on the side of the ptotic eye.

In our study, it was also revealed that eyebrow asymmetry could be present in normal individuals without eyelid ptosis (24.7% of patients in this study). This asymmetric brow height can lead to an asymmetric upper eyelid shape and contour. Aesthetic surgeons should be aware of this effect and avoid excessive skin removal in an eye with a lower brow, because it can create a false perception of more redundant skin.

Although in our study we demonstrated that eyebrow asymmetry is almost 3 times more prevalent in patients with ptosis compared with those without ptosis, we did not find an association between the severity of ptosis and the severity of eyebrow elevation. The general belief that eyebrow elevation on the side of ptosis is caused by visual deprivation is now being questioned by some authors.^12,13^ Sinha et al^14^ showed that this brow elevation in patients with dermatochalasis and ptosis does not necessarily change the MRD1. To show that the anatomical relationships between eyebrow and eyelid are not the cause of this compensatory mechanism, they mechanically elevated the eyebrow to the desired position and demonstrated that this maneuver was unable to change the eyelid position in patients with ptosis. Thus, they proposed a co-stimulation mechanism that couples the innervation of the frontalis and levator muscle. In this regard, Matsuo et al^15^ described a reflex contracture of the frontalis muscle in ptotic eyes caused by mechanoreceptor activation in Müller’s muscle that was stimulated by an elongation of the upper eyelid. This coupling theory is also partially inconclusive, because in our study we could not find any association between eyebrow height and severity of ptosis. Such association could have been found if there was a linear relationship between the level of mechanoreceptor stimulation and reflective frontalis contracture. Another point that this theory does not explain is the partial effect of ptosis correction on the eyebrow height change. Even though studies have shown that the mean eyebrow height decreases after successful levator resection and Muller's muscle conjunctival resection (MMCR) surgery, the results were unpredictable. In a study by Karlin and Rootman,^16^ brow asymmetry could be found in 50% of patients after MMCR. Furthermore, Kokubo et al^17,18^ stated that the magnitude of eyebrow changes occurring after ptosis surgery is not predictable, suggesting that other mechanisms or secondary forehead changes are involved in brow elevation. All things considered, in this study, we could not confirm the magnitude of linear eyebrow changes induced by eyelid ptosis severity, as revealed by Manta et al.^19^ Although in our study we can say that there was less chance of patients without asymmetry having significant ptosis and higher chance of those with severe eyebrow asymmetry having moderate ptosis, the difference was not statistically significant. One reason for this finding is that we did not have any patient with severe eyelid ptosis. Recruiting patients with severe ptosis could lead to a statistically significant difference in analyzing the effect of ptosis severity on brow asymmetry.

Finally, we identified ptosis in 13.7% of the individuals included in our study, which is significantly lower than the 57.6% reported by Chao et al.^20^ The notable disparity in participant numbers (59 vs 2530) and age distribution (63.7 vs 47.5 years) may account for this variation. In comparison with the Abdolalizadeh et al^5^ study with more than 1400 patients, the prevalence rate of ptosis in our study is very low (47% vs 13.7%); this discrepancy could be attributed to their assessment of dynamic ptosis in their study vs static ptosis in ours. Nevertheless, in both studies, the importance of identifying signs of masked ptosis prior to upper blepharoplasty is emphasized as a final conclusion.

We found several limitations in this study. First, ocular dominance as a disputable cause of eyebrow asymmetry was not sought. Even though the presence of eyebrow asymmetry could be the result of facial asymmetry, in patients without eyelid ptosis it could also be justified with the ocular dominance theory.^6^ Even though there are sufficient data against the effects of ocular dominance on brow asymmetry, we could neither support nor refute the effects of ocular dominance on our patients because of insufficient data. Additionally, we did not assess the severity of dermatochalasis to evaluate the impact of eyelid weight or visual dysfunction on the degree of brow elevation. This information would have been valuable in understanding the relationship between dermatochalasis and eyebrow asymmetry.

## Conclusions

It is important for aesthetic surgeons to recognize that involutional periocular changes can lead to undiagnosed eyelid ptosis, which may not be solely attributed to dermatochalasis. In this study, which can be considered the largest one that investigated ptosis prevalence in candidates for upper eyelid blepharoplasty, we found that ∼13% of these patients had undiagnosed ptosis. Although most of these cases were mild, it required some amount of caution on our part to detect them. The presence of eyebrow asymmetry could serve as an indicator for undiagnosed ptosis.
